# The Solastalgia Scale: A Turkish Validity and Reliability Study

**DOI:** 10.1192/j.eurpsy.2026.11101

**Published:** 2026-07-31

**Authors:** Ö. Yaman, A. İlez, F. Oflaz

**Affiliations:** 1 Psychiatric Nursing Depatrment; 2 Department of Psychiatric NursingPsychiatric Nursing Department, Koç University; 3 Department of Physiotherapy and Rehabilitation, Beykent University, Istanbul, Türkiye

## Abstract

**Introduction:**

Solastalgia refers to the place-based psychological distress individuals experience when their familiar environment is altered or degraded, despite no physical displacement. As ecological disruptions such as climate change and biodiversity loss intensify, solastalgia has emerged as a meaningful construct linking environmental degradation to emotional and mental health outcomes. However, research in non-Western contexts remains limited by the absence of culturally validated measurement tools.

**Objectives:**

This study aimed to adapt the Solastalgia subscale of the Environmental Distress Scale (EDS) into Turkish and evaluate its psychometric validity and reliability among Turkish-speaking adults.

**Methods:**

The adaptation process followed international standards including forward–backward translation, expert evaluation, and pilot testing. Data were collected from 439 Turkish-speaking adults. Exploratory and confirmatory factor analyses were conducted to assess construct validity. Internal consistency was evaluated using Cronbach’s alpha. Test-retest reliability and convergent validity with the Climate Change Worry Scale were also analyzed.

**Results:**

Psychometric analyses supported a strong unidimensional structure (explained variance = 53.5%), with item-total correlations ranging from 0.48 to 0.75. CFA indicated good model fit (χ^2^/df = 2.13, CFI = 0.974, RMSEA = 0.056). The scale demonstrated excellent internal consistency (Cronbach’s α = 0.92) and high test-retest reliability (r = 0.88, p < 0.001). Strong convergent validity was observed with the Climate Change Worry Scale (r = 0.82, p < 0.001). Solastalgia scores were significantly higher among participants with strong emotional bonds to nature, frequent engagement with natural environments, and greater ecological concern.

**Conclusions:**

The Turkish version of the Solastalgia Scale is a psychometrically sound tool for assessing emotional responses to environmental degradation. It holds significant potential for use in ecological psychology research, public health monitoring, and climate-related mental health interventions within culturally specific settings.

**Disclosure of Interest:**

None Declared

